# Landscape of associations between long non‐coding RNAs and infiltrating immune cells in liver hepatocellular carcinoma

**DOI:** 10.1111/jcmm.15690

**Published:** 2020-09-10

**Authors:** Li Li, Xiaowei Song, Yanju Lv, Qiuying Jiang, Chengjuan Fan, Dayong Huang

**Affiliations:** ^1^ Department of Oncology The Second Affiliated Hospital of Harbin Medical University Harbin China

**Keywords:** immune cell populations, immunity, liver hepatocellular carcinoma, long non‐coding RNA, prognosis

## Abstract

Liver hepatocellular carcinoma (LIHC) can be detected by the immune system; however, it acquires features for evasion of immune surveillance during its origin and development. Long non‐coding RNAs (lncRNAs) are critical as immune regulators in cancers; nevertheless, the biological functions and mechanisms of lncRNAs in evasion of immune system by LIHC remain unclear. In this study, an integrated and computational approach was developed to identify immune‐related lncRNAs and to divide LIHC patients into diverse immune‐related risk groups based on the expression profiles of coding genes and lncRNAs. LIHC‐specific genes and lncRNAs in 17 immune cell populations were identified and analysed. Gene and lncRNA co‐expressing networks for diverse immune cell populations were constructed and analysed. Some imported immune‐related lncRNAs, such as MIR9‐3HG, were also identified. The LIHC patients comprised three different groups based on immune‐related risk. LIHC patients possessing a greater diversity of immune cell populations had better survival prognosis. The collective data are evidence of a credible computational model that can prioritize novel immune‐related lncRNAs and depict the atlas of immune‐related lncRNAs in LIHC. These findings will further the understanding of lncRNA function and advance the identification of immunotherapy targets in LIHC.

## 1 INTRODUCTION

According to the International Agency for Research on Cancer, liver hepatocellular carcinoma (LIHC) is the third leading cause of cancer‐related death worldwide.^1^, ^2^ LIHC is also the sixth most diagnosed cancer, with more than 500 000 new cases reported every year.^3^ Continued research is revealing more risk factors of LIHC. These include hepatitis B virus infection, aflatoxin exposure, alcohol‐induced liver damage, non‐alcoholic fatty liver disease and non‐alcoholic steatohepatitis, among others.^4^, ^5^ Liver transplantation or curative surgery is effective for early stage LIHC. However, therapeutic strategies are limited for advanced cases.^6^ Despite the recent progress concerning diagnostic and therapeutic modalities, the survival rate of LIHC is still poor. Especially, tumour recurrence, drug resistance and disease relapse after treatment are still key problems that hinder LIHC therapy. There is an urgent need to improve LIHC diagnosis and treatment.

Long non‐coding RNAs (lncRNAs) are a subclass of functional non‐coding RNAs. LncRNAs are over 200 nucleotides in size and do not encode protein.^7^, ^8^ However, lncRNAs are reportedly essential in many diseases, including cancers.^9^ As well, lncRNAs have been associated with the regulation of biological functions of LIHC. For example, the up‐regulation of lncRNA DLX6‐AS1 can promote proliferation and tumour formation.^10^ The PCNAP1 lncRNA enhances replication of hepatitis B virus (HBV) by modulating microRNA‐154/proliferating cell nuclear antigen/HBV (miR‐154)/PCNA/HBV) covalently closed circular DNA signalling and PCNA pseudogene 1 (PCNAP1)/PCNA signalling drives LIHC.^11^ The up‐regulated expression of hepatocellular carcinoma up‐regulated EZH2‐associated (HEIH) lncRNA might inhibit the growth and metastasis of LIHC cells.^12^ While accumulating evidence indicates the important functions of lncRNA in LIHC, the systematic mechanism and function of lncRNAs in LIHC are poorly defined.

The tumour microenvironment is considered a key risk factor for cancer development and progression. Immune system imbalance and disorders are major causes of cancer.^13^ Tumours can be detected by the immune system, and they acquire features to evade immune surveillance throughout the origin and development of cancer. Immunotherapy has emerged as a promising cancer treatment strategy. Dysregulation of immune genes could directly influence the immune system. Recent increasing evidence has indicated the participation of lncRNAs in the immune system.^14^, ^15^ For example, inducible degradation of the Sros1 lncRNA promotes interferon‐gamma‐mediated activation of innate immune responses by stabilizing Stat1 mRNA.^16^ The HOTTIP lncRNA enhances interleukin 6 expression to potentiate immune escape of ovarian cancer cells by up‐regulating the expression of programmed death‐ligand 1 in neutrophils.^17^ However, data concerning the diversity and function of immune‐related lncRNAs in cancer are insufficient. Further studies of lncRNAs and their roles in immune regulation are essential to identify immunotherapy targets in LIHC.

In the present study, an integrated computational pipeline was developed based on gene and lncRNA expression profiles to identify immune‐related lncRNAs in LIHC. LIHC patients were divided into three immune‐related risk groups. We constructed 17 sets of immune cell populations and identified differentially expressed genes in these sets. Diverse immune cell‐related genes and lncRNA co‐expressed networks were constructed and analysed. Some imported immune‐related lncRNAs, such as MIR9‐3HG, were also identified. LIHC patients harbouring more diverse immune cell populations displayed a better survival prognosis. The data will provide a valuable resource for exploration of lncRNA function and identification of immunotherapy targets in LIHC.

## 2 MATERIAL AND METHODS

### 2.1 Collection of high‐throughput expression profiles of lncRNAs and genes for LIHC

Gene and lncRNA expression profiles (Level 2) were obtained from The Cancer Genome Atlas (https://www.cancer.gov/about-nci/organization/ccg/research/structural-genomics/tcga). LIHC patients (n = 369) and 50 tumour adjacent normal tissues were obtained. Clinical follow‐up information was retained for further analysis. Genes and lncRNAs with expression values of 0 in the samples were removed. All expression values were log_2_ transformed to satisfy normal distribution. The summary of characters and clinical follow‐up information for LIHC patients and normal control was listed in Table S1.

### 2.2 Collection of multiple immune cell populations‐related (ICPR) genes

Seventeen immune cell populations were selected. They included B cells, eosinophils, macrophages, mast cells, natural killer (NK) CD56bright cells, NK CD56dim cells, neutrophils, T helper cells, Tcm cells, Tem cells, Tfh cells, activated dendritic cells (aDCs), induced dendritic cells (iDCs), activated CD8 T cells, gamma delta T cells, regulatory T cells (Tregs) and cytotoxic cells.^18^ Information on multiple ICPR genes was also obtained from previous studies.^19^, ^20^ All the ICPR genes were analysed.

### 2.3 Identification of LIHC‐specific ICPR genes and lncRNAs based on expression profiles

In order to identify LIHC‐specific ICPR genes and lncRNAs, the t test was used for differential expression analyses of the expression profiles of LIHC and normal samples. Significantly expressed ICPR genes (*P* < .05) were considered LIHC‐specific ICPR genes. Significant lncRNAs (*P* < .01) were considered LIHC‐specific lncRNAs. Only the LIHC‐specific ICPR genes and lncRNAs were used for subsequent analyses.

### 2.4 Construction of ICPR genes and lncRNAs associated with significantly changed co‐expressed networks for each immune cell population in LIHC

For each immune cell population, Pearson correlation coefficients (PCCs) were calculated for each LIHC‐specific ICPR gene and lncRNA in LIHC and normal samples, respectively. PCCs and *P*‐values were obtained for each ICPR gene and lncRNA pair. The absolute values of the difference of PCCs between LIHC and normal samples represented the divergence of LIHC compared with normal samples. Only the LIHC‐specific ICPR gene and lncRNA pairs with absolute values of difference >0.5 were used for subsequent analyses and network construction. The significantly changed co‐expressed networks of LIHC‐specific ICPR genes and lncRNAs were constructed using Cytoscape 3.3.0 (https://cytoscape.org/). Degree analysis was also performed using Cytoscape 3.3.0.

### 2.5 Evaluating immune‐associated risks for LIHC patients based on co‐expressed networks

**FIGURE 1:**
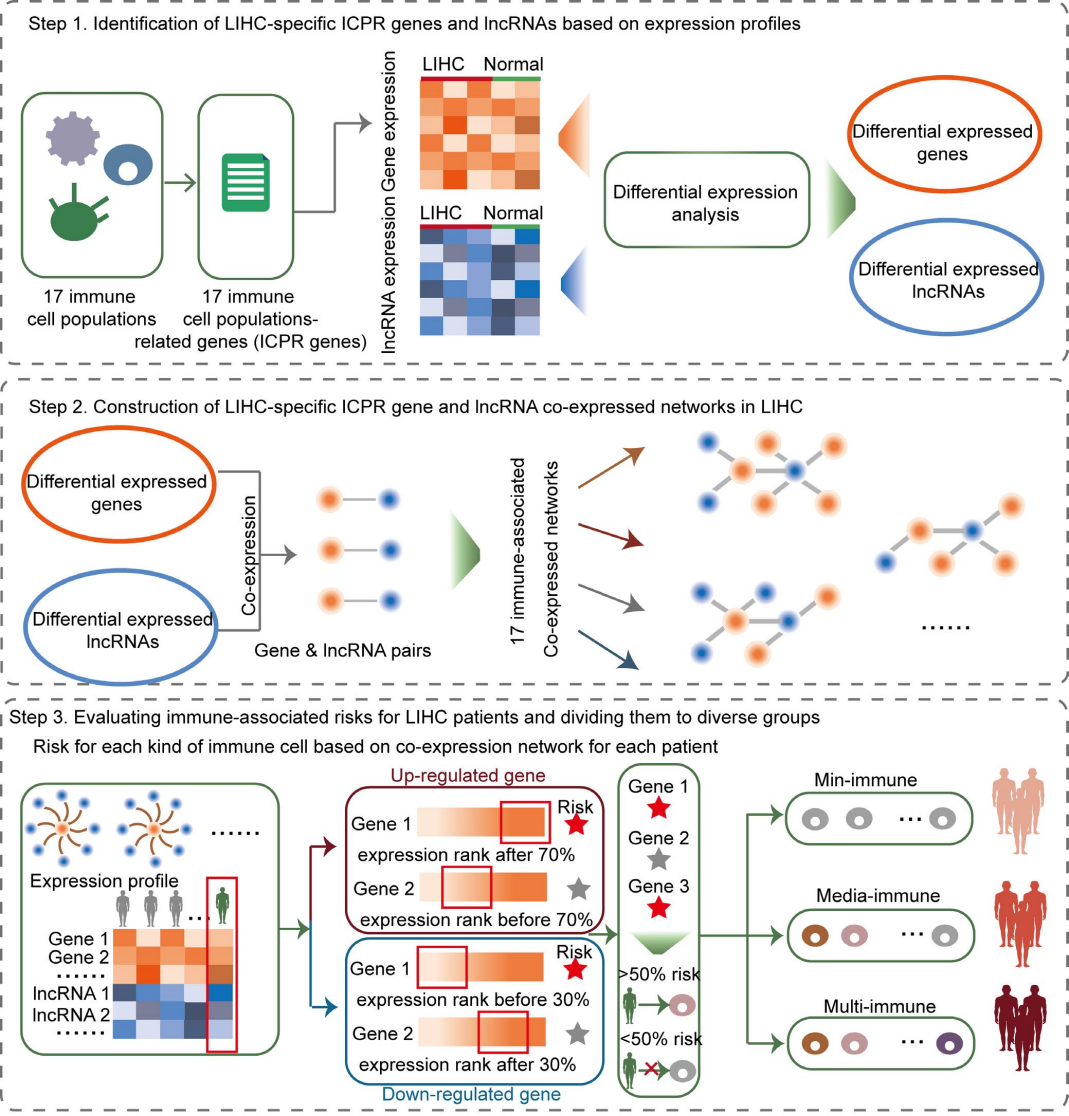
Workflow of evaluating the immune risk for LIHC patients based on ICPR genes and lncRNAs. Step 1: Identification of LIHC‐specific ICPR genes and lncRNAs based on expression profiles. Step 2: Construction of LIHC‐specific ICPR genes and lncRNAs co‐expression networks for diverse kinds of immune cell populations. Step 3: Evaluating immune risk for LIHC patients and dividing the patients into groups

An integrated and computational pipeline was developed to evaluate immune‐associated risks for each LIHC patient based on co‐expressed networks (Figure 1). Firstly, the expression profiles of genes and lncRNAs in co‐expressed network for each immune cell population were extracted from the whole expression profile. The differentially expressed genes and lncRNAs were allocated to up‐regulated and down‐regulated groups. Secondly, the expression values were ranked from high‐to‐low for all LIHC patients. For up‐regulated genes and lncRNAs, the genes or lncRNA of a specific sample ranked last 70% were considered risk genes or lncRNAs for the LIHC patient. For down‐regulated genes and lncRNAs, the genes or lncRNA of a specific sample ranked before 30% were considered risk genes or lncRNAs for the LIHC patient. Thirdly, for a specific LIHC patient, the risk genes in each kind of ICPR co‐expression network were counted. If there were more than 50% risk genes or lncRNAs for an immune cell population, this population was considered a risk immune cell population for that LIHC patient. Lastly, the numbers of risk immune cell populations were counted and the LIHC patients were divided into three immune risk‐related groups including min‐immune (0‐4 risk immune cell populations), media‐immune (5‐9 risk immune cell populations) and multi‐immune groups (10‐17 risk immune cell populations).

### 2.6 Survival analyses for multiple kinds of immune risk‐related groups

In order to evaluate the prognosis of the three immune risk‐related groups, Kaplan‐Meier (K‐M) survival analyses were performed. Statistical significance assessed using the log‐rank test. All analyses were performed within the R 2.6.6 framework.

## 3 RESULTS

### 3.1 Some ICPR genes and lncRNAs were specific in LIHC

**FIGURE 2:**
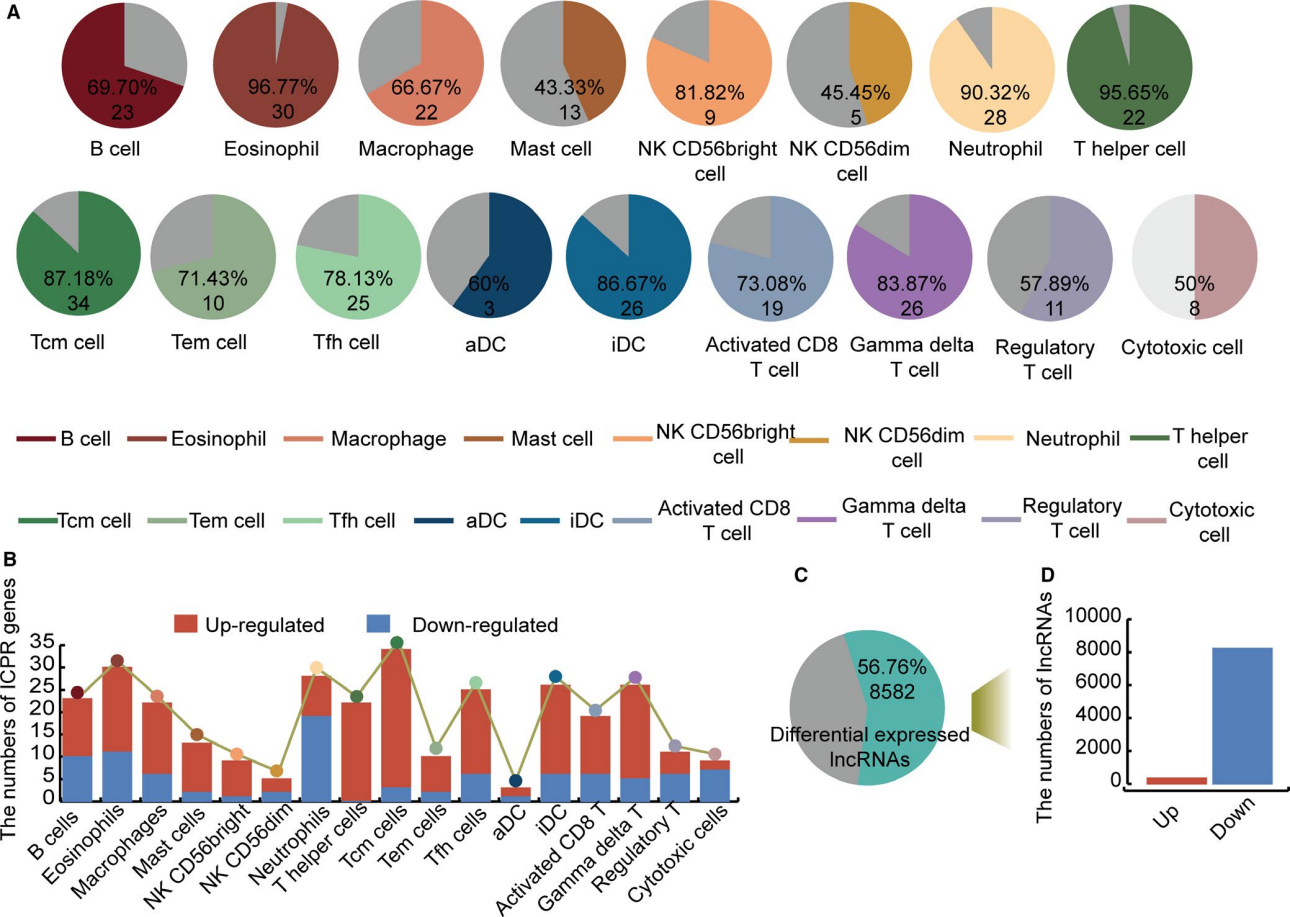
Identification of LIHC‐specific ICPR genes and lncRNAs for different immune cell populations. A, Pie charts show the percentages of significantly differentially expressed ICPR genes and lncRNAs for diverse immune cell populations in LIHC. B, Bar plots show numbers of up‐ (red) and down‐regulated (blue) ICPR genes in LIHC. C, Pie chart shows percentage of significantly differentially expressed lncRNAs in LIHC. D, Bar plots show numbers of up‐ (red) and down‐regulated (blue) lncRNAs in LIHC

We obtained ICPR gene information for the 17 immune cell populations and explored their specificity in LIHC. Differentially, expressed ICPR genes were identified for each immune cell population in the LIHC patients (Figure 2A). These were considered LIHC‐specific ICPR genes. The differentially expressed ICPR genes accounted for a large proportion of most of the immune cell populations. The proportions of LIHC‐specific ICPR genes were 96.77%, 95.65% and 90.32% in eosinophils, T helper cells and neutrophils, respectively. These results indicated that multiple types of immune cells may have essential roles in LIHC. Up‐ and down‐regulated ICPR genes were also identified (Figure 2B). The vast majority of LIHC‐specific ICPR genes were down‐regulated in most of the immune cell populations. For example, all the LIHC‐specific ICPR genes were down‐regulated in T helper cells. There were 13 down‐ and six up‐regulated ICPR genes in activated CD8 T cells, and 19 up‐ and nine down‐regulated ICPR genes in neutrophils. Differentially expressed lncRNAs, considered as LIHC‐specific lncRNAs, were also identified (56.76%) (Figure 2C). Similar to LIHC‐specific ICPR genes, most of the differentially expressed lncRNAs were down‐regulated (Figure 2D). These results revealed that immune‐related genes and some lncRNAs may participate in the development of LIHC.

### 3.2 Significant change in co‐expression between LIHC‐specific ICPR genes and lncRNAs in LIHC compared with normal samples

**FIGURE 3:**
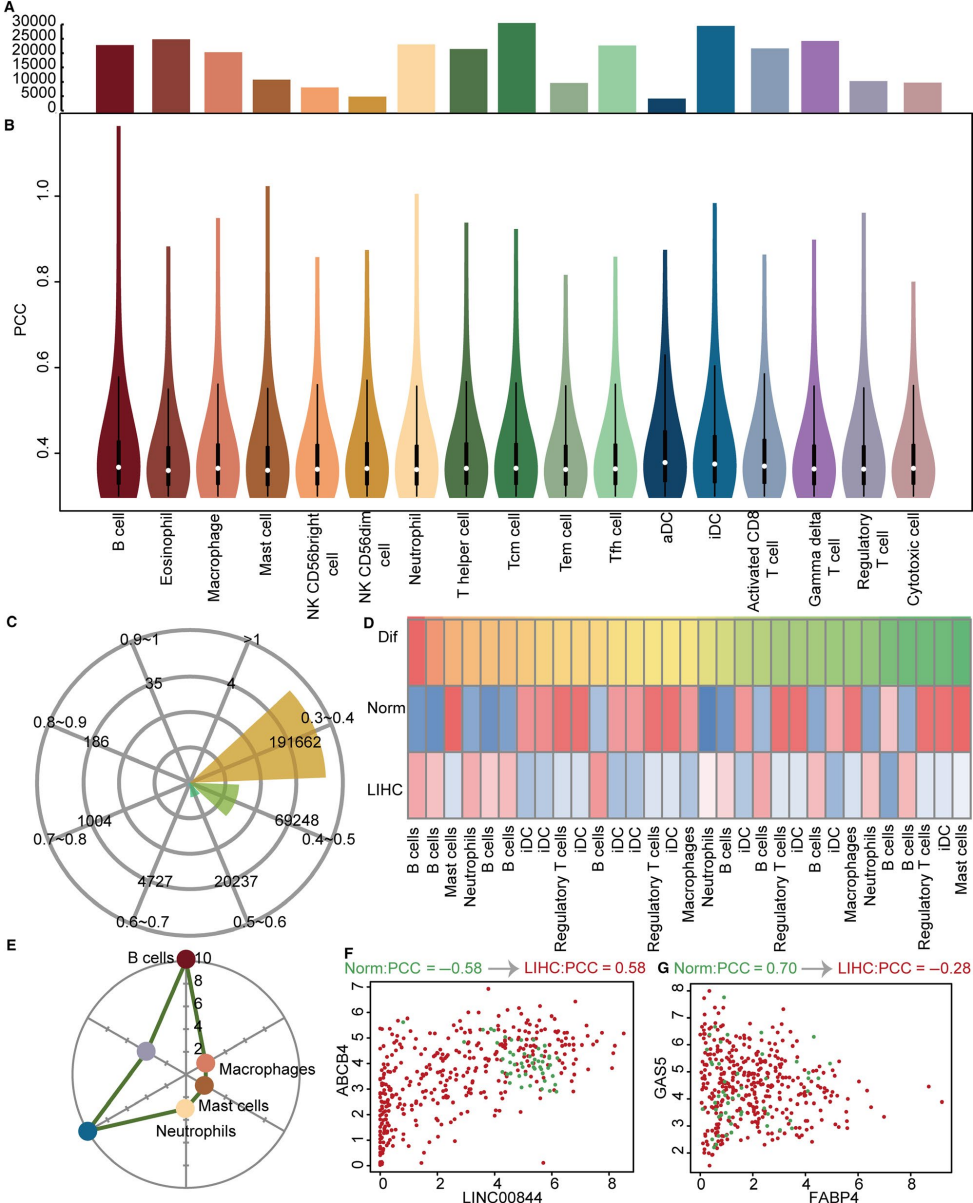
LIHC‐specific ICPR gene and lncRNA co‐expressed pairs. A, Bar plots show numbers of LIHC‐specific ICPR gene and lncRNA co‐expressed pairs in diverse immune cell populations. B, Vioplots show PCCs of LIHC‐specific ICPR gene and lncRNA co‐expressed pairs in diverse kinds of immune cell populations. C, Rose diagram shows numbers of LIHC‐specific ICPR gene and lncRNA co‐expressed pairs with different PCCs. D, Heat map shows PCCs of top LIHC‐specific ICPR gene and lncRNA co‐expressed pairs for LIHC, normal samples, and difference. Red and blue represent higher and lower PCCs. E, Radar diagram shows numbers of top pairs in diverse kinds of immune cell populations. F, G, Points plots show expression of lncRNA (*X* axis) and gene (*Y* axis). Red and green represent LIHC and normal samples

We used the absolute difference of PCCs for ICPR genes and lncRNAs between LIHC patients and normal control samples to represent the changes in LIHC. We only considered LIHC‐specific ICPR gene and lncRNA pairs having absolute difference values >0.3. The numbers of co‐expressed LIHC‐specific ICPR gene and lncRNA pairs differed in the diverse immune cell populations. Tcm cells and aDCs displayed the most and least LIHC‐specific ICPR gene and lncRNA pairs, respectively (Figure 3A). The distribution patterns of PCCs for LIHC‐specific ICPR gene and lncRNA pairs were different in the diverse immune cell populations which were similar (Figure 3B). Most PCCs for LIHC‐specific ICPR gene and lncRNA pairs were concentrated between 0.3 and 0.6. Of these, 191 622 were concentrated between 0.3 and 0.4 (Figure 3C) and 69 248 were concentrated between 0.4 and 0.5. Thirty LIHC‐specific ICPR gene and lncRNA pairs displayed the most significant differences (Figure 3D). There were two patterns of change in samples from LIHC patients compared with normal samples. One was a negative to positive correlation, and the other was a positive to negative correlation. The patterns of change for these top 30 pairs were diverse. These pairs were prominent in six immune cell populations (Figure 3E). Twenty of the LIHC‐specific ICPR gene and lncRNA pairs belonged to B cells and iDCs. In B cells, ICPR gene ABCB4 and lncRNA were negatively correlated (PCC = −0.58) in normal control samples. However, the ICPR gene ABCB4 and lncRNA LINC00844 changed to a positive correlation (PCC = 0.58) in LIHC patients (Figure 3F). The changed pattern was contrary for ICPR gene ABCB4 and lncRNA FABP4, with a change in PCC of 0.70 in normal samples to −0.28 in LIHC patients (Figure 3G). The collective results indicated a changed co‐expression of ICPR genes and lncRNAs in LIHC, which reflected immune changes of lncRNAs in LIHC.

### 3.3 Particular features of co‐expressed networks of LIHC‐specific ICPR genes and lncRNAs

**FIGURE 4:**
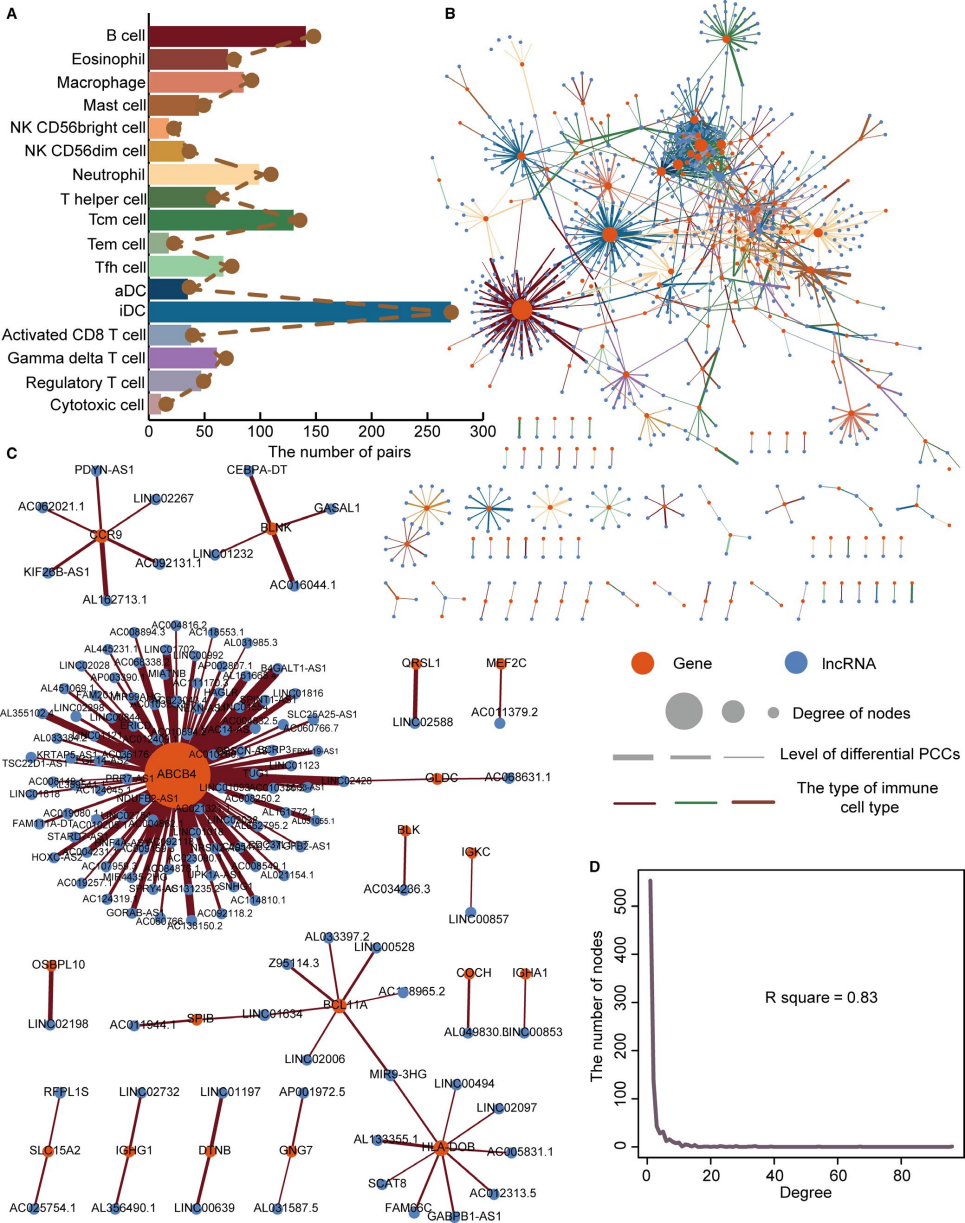
Construction of LIHC‐specific ICPR gene and lncRNA co‐expressed networks in diverse kinds of immune cell populations. A, Bar plots show numbers of pairs of networks in diverse kinds of immune cell populations. B, Integrated LIHC‐specific ICPR gene and lncRNA co‐expressed network. C, LIHC‐specific ICPR gene and lncRNA co‐expressed network in B cells. D, Degree distribution of integrated LIHC‐specific ICPR gene and lncRNA co‐expressed network

In order to better describe the role of immune changes in LIHC, more significant changes (absolute difference value >0.7) for LIHC‐specific ICPR gene and lncRNA PCCs were used to construct networks. The numbers of LIHC‐specific ICPR gene and lncRNA pairs in each immune cell population were determined (Figure 4A). iDCs and B cells displayed the highest number of LIHC‐specific ICPR gene and lncRNA pairs. A comprehensive network of LIHC‐specific ICPR gene and lncRNA pairs was constructed by integrating the multiple immune cell populations (Figure 4B). The integrated network revealed obvious differences between the diverse LIHC‐specific ICPR gene and lncRNA pairs. The same ICPR gene could also interact with different lncRNAs with diverse strengths and changing patterns. Only a few ICPR genes were prominent. These may be important in LIHC. A similar network was also constructed in single immune cell population. For example, a co‐expressed network of LIHC‐specific ICPR genes and lncRNAs was constructed in B cells (Figure 4C). This network contained 141 pairs of LIHC‐specific ICPR genes and lncRNAs, 18 ICPR genes and 138 lncRNAs. The integrated degree distributions of the network displayed a scale‐free network power‐law distribution (*R* ^2^ = .83, Figure 4D). This scale‐free distribution indicated that this network was a meaningful biological network.

### 3.4 Identification of important ICPR genes and corresponding lncRNAs in LIHC

**FIGURE 5:**
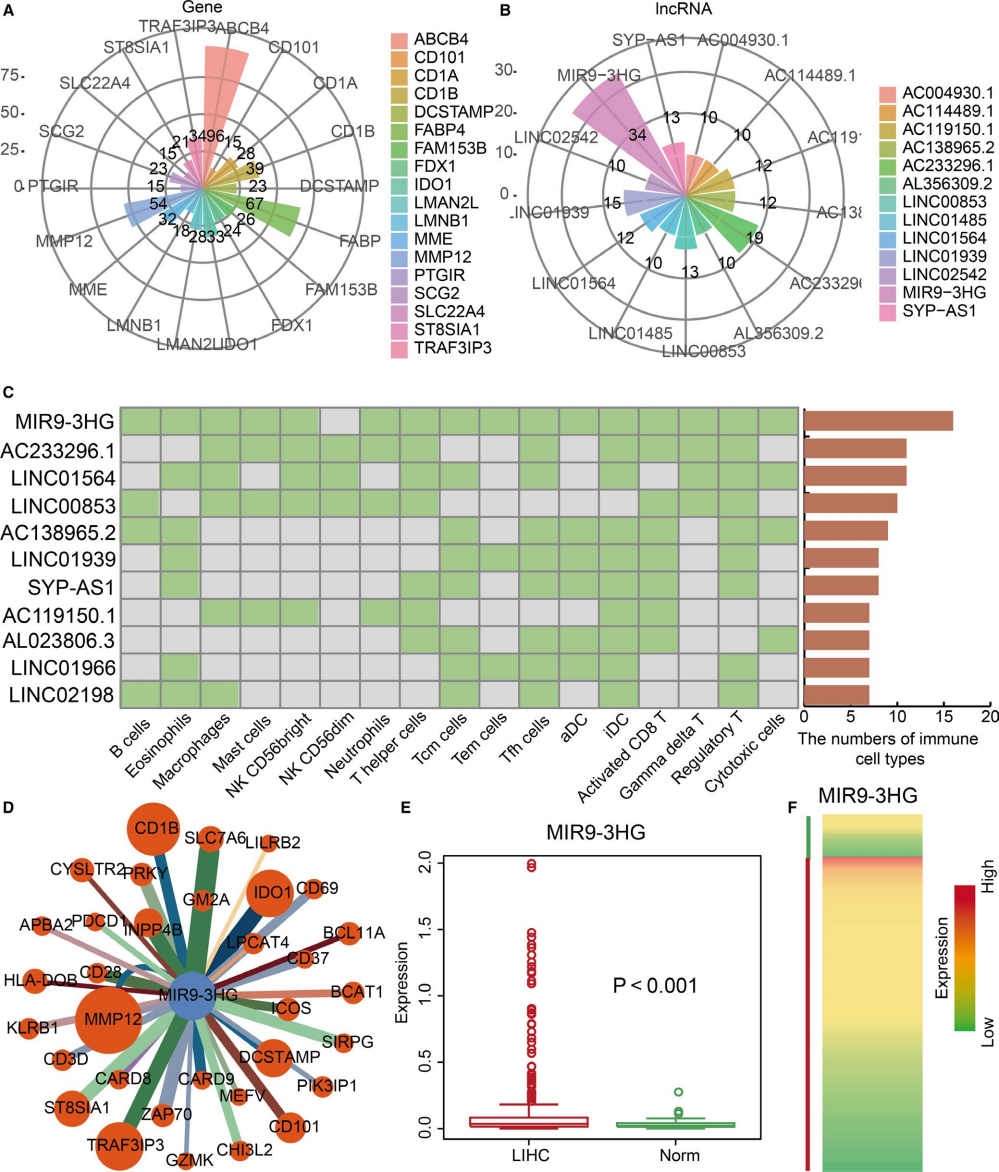
Identification and characteristics of key LIHC‐specific ICPR genes and lncRNAs. A, Rose diagram shows the degree of key LIHC‐specific immune‐related lncRNAs. B, Rose diagram shows the degree of key LIHC‐specific lncRNAs. C, Heat map shows immune cell populations which are associated with immune‐related lncRNAs in LIHC. D, MIR9‐3HG centric network in all the immune cell populations. E, Box plot shows expression of MIR9‐3HG in LIHC (red) and normal samples (green). F, Heat map shows expression of MIR9‐3HG

Some ICPR genes and corresponding lncRNAs were more frequently present in the aforementioned co‐expression networks of LIHC‐specific ICPR genes and lncRNAs. Thus, we inferred that at least some of these frequent ICPR genes and corresponding lncRNAs may play important roles in LIHC. These ICPR genes included ABCB4, CD101, CD1A, CD1B and FABP4 (Figure 5A). ABCB4 is an immune‐related gene as well as a phospholipid translocator at the canalicular membrane of the hepatocyte, which "flops" phosphatidylcholine into bile.^21^ In our study, ICPR gene ABCB4 interacted with 96 lncRNAs in LIHC, indicating immune‐related roles in concert with other lncRNAs in LIHC. More attention was paid to the frequent lncRNAs, which were considered to be immune‐related lncRNAs in LIHC. Other immune‐related lncRNAs that were frequent included MIR9‐3HG, SYP‐AS1 and LINC02542 (Figure 5B). These immune‐related lncRNAs were present in different immune cell populations (Figure 5C). For example, MIR9‐3HG was present in all immune cell populations, except NK CD56dim, in LIHC patients. MIR9‐3HG has been associated with some cancers.^22^, ^23^ MIR9‐3HG was presently co‐expressed with 34 different ICPR genes in LIHC (Figure 5D). Major changes were observed in some of these interactions, including TRAF3IP3 and SLC7A6. Small changes were also evident, such as for GZMK and PIK3IP1. MIR9‐3HG was significantly differentially expressed (*P* < .001) in LIHC compared with normal control tissues (Figure 5E). The expression of MIR9‐3HG was higher in LIHC tissues (Figure 5F). These collective results revealed essential roles of some lncRNAs in the regulation of the immune system of LIHC.

### 3.5 Different survival outcomes in LIHC for the diverse immune risk groups

**FIGURE 6:**
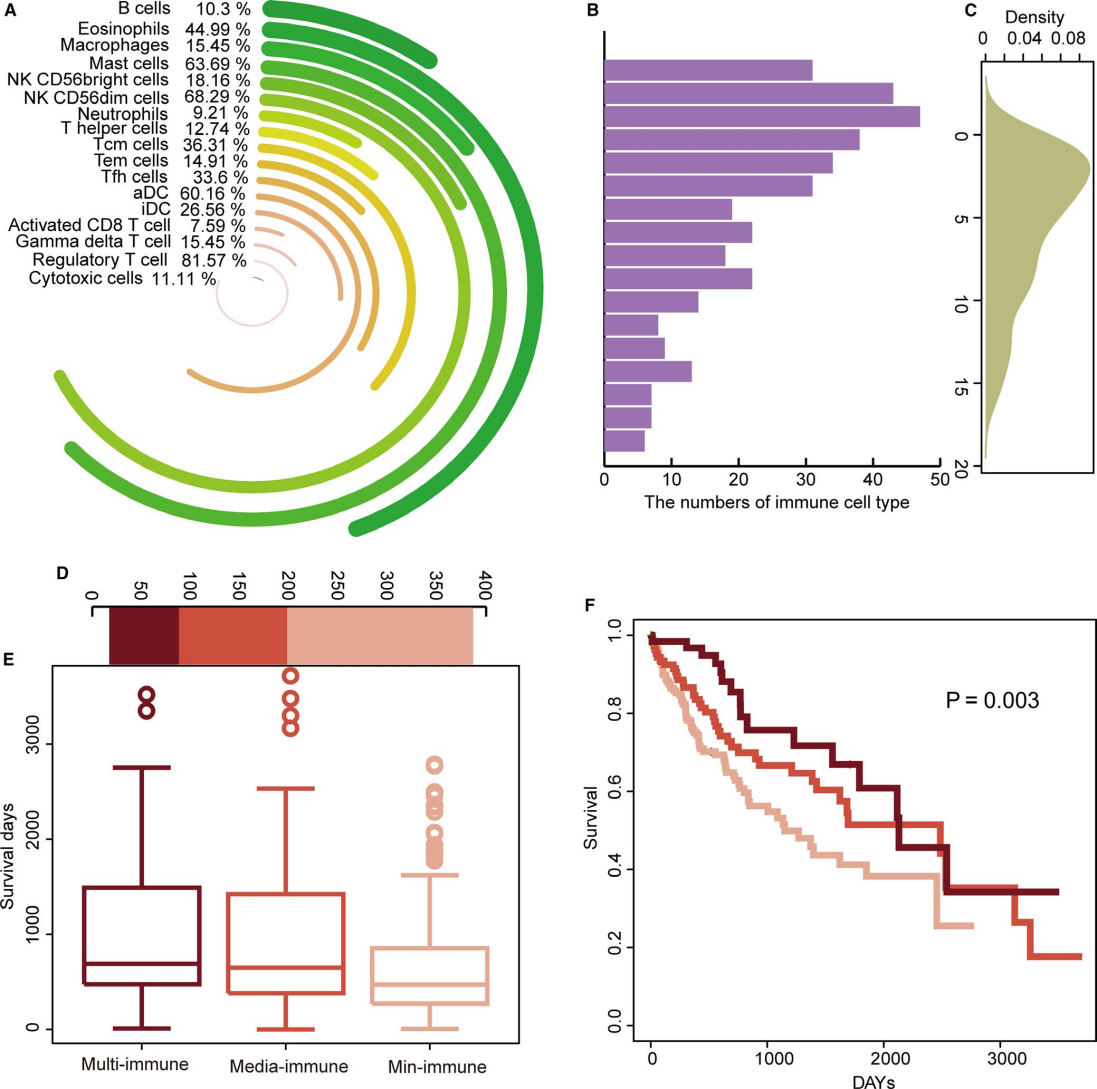
Diverse immune risk groups are associated with survival. A, Circle diagrams show percentages of LIHC samples related to diverse kinds of immune cell populations. B, Bar plot shows numbers of samples in different numbers of immune cell populations. C, Density plot shows distribution of samples with different numbers of immune cell populations. D, Numbers of LIHC samples in the diverse immune risk groups. E, Box plots show survival days of diverse immune risk groups. F, Survival plot shows prognosis of diverse immune risk groups

Evaluating the immune risk of each LIHC patient could be beneficial in clarifying patient status and for personalized treatment. We inferred that LIHC patients harbouring more diverse immune cell populations could be considered higher immune risk patients. The LIHC patients differed in their diversity of immune cell populations (Figure 6A). For example, 81.57% and 68.29% of the LIHC patients were associated with Treg and NK CD56dim cells, respectively. However, only 9.21% of the LIHC patients were related to neutrophils. We also analysed the numbers of immune cell populations for each LIHC patient. Forty‐seven LIHC patients harboured two kinds of immune cells populations and 43 harboured one kind (Figure 6B). Only a few LIHC patients harboured multiple immune cell populations (Figure 6C). The data were used to divided the LIHC patients into three immune risk groups of multi‐immune (n = 64), media‐immune (n = 110) and min‐immune groups (n = 189; Figure 6D). Survival increased as the immune risk decreased (Figure 6E). The survival analysis also revealed that diverse immune risk groups were significantly associated with survival (*P* = .003, Figure 6F). More immune risk types for a LIHC patient implied stronger immune participation in the specific sample. Thus, a specific LIHC patient would have a better prognosis with the alteration of multiple immune cell populations in LIHC.

## 4 DISCUSSION

Accumulating evidence is reinforcing the importance of lncRNAs as immune regulators. However, only a few examples had been found so far. Here, we describe an integrated computational approach to identify immune‐related lncRNAs in LIHC based on expression profiles of ICPR genes and lncRNAs. We demonstrated that lncRNA are essential in the immune regulation of LIHC. Co‐expression networks of ICPR genes and lncRNAs for 17 immune cell populations were constructed and analysed in LIHC. Notably, each LIHC patient was given an immune risk score based on diverse immune cell populations. All the LIHC patients were divided into diverse immune risk groups, and they showed different prognoses.

Liver hepatocellular carcinoma is highly heterogeneous immunologically, and immune‐related genes have been identified and studied.^24^, ^25^ Most of these data involve coding genes. Some important non‐coding RNAs, including lncRNAs, have been ignored. LncRNAs are emerging as critical regulators of gene expression and play fundamental roles in immune regulation for many kinds of cancers.^15^ In the present study, we integrated gene and lncRNA expression in order to globally describe immune characteristics for LIHC patients. We discovered that some of the identified immune‐related genes and lncRNAs were associated with immune functions and cancer in previous experiments (Table S2). Some key immune‐related lncRNAs, such as MIR9‐3HG, SYP‐AS1 and LINC02542, were identified. LINC01564 could interact with 12 ICPR genes and was considered a cancer‐related lncRNA.^26^ More significantly, LINC01564 was also successfully detected in patient urine samples and was up‐regulated when compared with urine from normal (healthy) individuals. These immune‐related lncRNAs were co‐expressed with many ICPR genes in LIHC. They may regulate ICPR gene expression or may work with ICPR genes together in LIHC. Presently, a co‐expression network was used to identify immune‐related lncRNAs. Weighted correlation network analysis (WGCNA) is also an effective method to determine relationships between genes and lncRNAs. WGCNA retains the continuity of network node connectivity and can identify core modules. However, different data preprocessing and analysis parameters will also lead to different results for WGCNA. Thus, appreciable differences can be evident for selected core modules. With a small number of known immune genes, comparing to WGCNA the co‐expressed network may contain more global information. In future studies, we intend to investigate the differences in the co‐expression network and WGCNA approaches in the identification of immune‐related lncRNAs.

The tumour microenvironment can be classified into immunologically active “inflamed” tumours and inactive “non‐inflamed” tumours based on the infiltration of cytotoxic immune cells.^24^ More precise classification of the LIHC microenvironment could improve our understanding of its immune diversity and immunotherapeutic response. Thus, dividing LIHC patients into diverse groups based on immune risk could be beneficial for diagnosis, prognosis and treatment. Presently, the LIHC patients were divided into three immune risk groups. Those patients harbouring more types of immune cell populations had better survival outcomes. Other classification methods produced consistent performance. For example, dividing the samples into two groups with seven as the boundary value yielded significant prognosis results (*P* = .02), as did dividing the samples into two groups with six as the boundary value (*P* = .02). These results indicate that patients with more immune cell types tend to have a better prognosis.

There are many immune cell populations in immune systems. They communicate with each other and also have their own functions. Most previous studies only explored the influence of the immune system on patients, without considering the various populations of immune cells. Presently, we defined 17 immune cell populations and analysed their respective characteristics in LIHC. Diverse LIHC patients were associated with different immune cell populations. Some immune cell populations, such as Treg cells, were present in more than 80% of the LIHC patients. However, activated CD8 T cells were only present in 7.59% of the samples. The present study provides a standardized computational pipeline to identify immune‐related lncRNAs in LIHC. It also provides a data resource concerning candidate immune‐related lncRNAs for further analyses in LIHC. Future studies will focus on investigating more LIHC samples to validate the accuracy and stability of the method presented here. Experimental validation of our results is also necessary.

In summary, lncRNAs are an important layer in the immune complexity in LIHC. Dividing LIHC patients into diverse immune risk groups would aid in evaluating prognosis. Our study provides an insight for the identification of novel immune‐related biomarkers and immunotherapies for LIHC.

## CONFLICT OF INTEREST

The authors declare that they have no competing interests.

## AUTHOR CONTRIBUTIONS

**Li Li:** Formal analysis (equal); Methodology (equal). **Xiaowei Song:** Data curation (equal); Formal analysis (equal); Methodology (equal). **Yanju Lv:** Software (equal); Visualization (equal). **Qiuying Jiang:** Software (equal); Validation (equal). **Chengjuan Fan:** Validation (equal). **Dayong Huang:** Conceptualization (lead); Writing‐original draft (lead); Writing‐review & editing (lead).

## ETHICS APPROVAL AND CONSENT TO PARTICIPATE

Not applicable.

## PATIENT CONSENT FOR PUBLICATION

Not applicable.

## CONSENT FOR PUBLICATION

Not applicable.

## Supporting information

Table S1

Table S2

## Data Availability

The data that support the findings of this study are available from the corresponding author upon reasonable request.
